# PITCH (pediatric infratentorial tumors – hydrocephalus-related complications) registry study design: observational, prospective, multicenter study evaluating the number of surgeries associated with the treatment of hydrocephalus secondary to infratentorial tumors in childhood and adolescence

**DOI:** 10.1007/s00381-026-07282-0

**Published:** 2026-05-08

**Authors:** Marcos Devanir Silva da Costa, Paloam Cardoso Nôvo, Thaís Neri Andrade de Almeida Garcia, Guilherme Ávila Girotto de Camargo, Bruna de Ávila Medeiros, Paulo Ronaldo Jube-Ribeiro, Carlos Eduardo Barros Jucá, Benicio Oton de Lima, Márcio Ferreira Marcelino, Tatiana Protzenko Cervante, Giovani Mendes Ferreira, Pedro Tadao Hamamoto Filho, Marcelo Volpon Santos, Gustavo Botelho Sampaio, Igor Vilela Faquini, Mariela Cecilia Salerno, Ramiro del Rio, Esdras Ismael Borrayo Flores, Simone Mendes Rogério, Vitor Nagai Yamaki, Emilio Pelleriti, Sandrieli Afornali, Carlos Alberto Mattozo, Adriano Keijiro Maeda, Eduardo Cortés Silva, Ricardo de Amoreira Gepp, Jorge Wladimir Junqueira Bizzi, Alexandre Varella Giannetti, Cleiton Formentin, Roberto Alexandre Dezena, Gabriel Mufarrej, Wagner Lazaretto Padua, Thais Cristina de Souza Melo, Fernando Seiji Suzuki, Patrícia Alessandra Dastoli, Sergio Cavalheiro

**Affiliations:** 1 Department of Neurology and Neurosurgery, Universidade Federal de Sao Paulo (Unifesp), Rua Napoleão de Barros, 715, 6th Floor, 04024-002 Sao Paulo, SP Brazil; 2 Complexo Oncológico de Referência de Goiás (CORA), Goiânia, Brazil; 3 Hospital Infantil Albert Sabin, Fortaleza, Brazil; 4 Hospital da Criança de Brasília, Brasília, Brazil; 5 Instituto Fernandes Figueira (IFF/Fiocruz), Rio de Janeiro, Brazil; 6 Hospital Santa Rosa, Cuiabá, Brazil; 7 Hospital das Clinicas da Faculdade de Medicina de Botucatu, Universidade Estadual Paulista, UNESP, Botucatu, Brazil; 8 Hospital das Clínicas da Faculdade de Medicina de Ribeirão Preto da USP - HCFMRP/USP, Ribeirão Preto, Brazil; 9 Hospital da Criança E Maternidade de São José Do Rio Preto, São José Do Rio Preto, Brazil; 10 Hospital da Restauração/IMIP, Instituto de Medicina Integral de Pernambuco, Recife, Brazil; 11 Hospital de Niños Sor Maria Ludovica, Buenos Aires, Argentina; 12 Hospital de Pediatría Juan P. Garrhan, Buenos Aires, Argentina; 13 Hospital El Pilar E Asociación Española de Beneficencia, Guatemala City, Guatemala; 14 Hospital Oncológico Infantil Octávio Lobo, Belém, Brazil; 15 Hospital Pediátrico Dr. Humberto Notti, Mendonza, Argentina; 16 Hospital Pequeno Príncipe, Curitiba, Brazil; 17 Hospital San Vicente Fundación, Medellín, Colombia; 18 Rede SARAH de Hospitais de Reabilitação, Brasília, Brazil; 19 Hospital da Criança Santo Antônio da Santa Casa de Porto Alegre, Porto Alegre, Brazil; 20 Faculdade de Medicina da Universidade Federal de Minas Gerais, Belo Horizonte, Brazil; 21 Centro Infantil Boldrini, Faculdade de Ciências Médicas da Universidade Estadual de Campinas, UNICAMP, Campinas, Brazil; 22 Universidade Federal Do Triângulo Mineiro (UFTM), Uberaba, Brazil; 23 Instituto Estadual Do Cérebro Paulo Niemeyer (IEC), Rio de Janeiro, Brazil

**Keywords:** Endoscopic third ventriculostomy, Ventriculoperitoneal shunt, External ventricular drainage, Treatment outcomes

## Abstract

**Introduction:**

Posterior fossa tumors are the most common solid pediatric neoplasms, and more than 60% of these tumors are associated with hydrocephalus, which can be managed using different strategies, including endoscopic third ventriculostomy (ETV), ventriculoperitoneal shunt (VPS), external ventricular drainage (EVD), or direct tumor resection without CSF diversion. The safest and most effective drainage method remains controversial, and most available studies are limited to single-center retrospective analyses, often constrained by small sample sizes. Therefore, multicenter prospective studies are needed to determine the optimal treatment strategy.

**Methods:**

This is a prospective, multicenter cohort study conducted across more than 20 pediatric neurosurgery centers in Latin America. Patients will be allocated into four groups according to the treatment selected for hydrocephalus (ETV, EVD, VPS, or resection). The primary outcome will be the number of surgical interventions related to the treatment of hydrocephalus during the follow-up period. Secondary outcomes will include mortality, infection, and other clinically relevant complications, analyzed as complementary endpoints. Patients will be followed prospectively for up to 12 months after the index procedure, defined as the intervention performed for the treatment of hydrocephalus, and each group will include at least 50 patients.

**Conclusion:**

The REDCap online platform will be used for data collection in the PITCH study, enabling prospective data acquisition across multiple centers in Latin America. This will allow comparison of treatment modalities for obstructive hydrocephalus secondary to posterior fossa tumors (ETV, EVD, VPS, and resection) and evaluation of their impact during the first year after diagnosis.

## Introduction

Among all childhood cancers, central nervous system (CNS) tumors account for 20.7%, second only to leukemias; however, among solid tumors, they are the most common, and of these, infratentorial tumors represent 60% of cases [1]. Posterior fossa tumors are associated with hydrocephalus [2], specifically non-communicating hydrocephalus, since most of these tumors cause direct or indirect obstruction of the fourth ventricle and/or its outflow pathways [3–5].

In a study of 117 children with posterior fossa tumors, Culey et al. [4] reported hydrocephalus in 83% of the patients. Dias MS et al. [5] concluded that one-quarter to one-third of infants with posterior fossa tumors were associated with hydrocephalus requiring diversion.

Although it may be intuitive to think that obstructive hydrocephalus can be treated with tumor resection, several factors predispose to persistent hydrocephalus after tumor removal: young age, preoperative hydrocephalus, midline tumor location, metastases, subtotal resection, tumor pathology, use of external ventricular drains, and postoperative complications [6].

There are several clinical contexts related to the treatment of hydrocephalus secondary to posterior fossa tumors; therefore, various management strategies for hydrocephalus secondary to infratentorial tumors have been described, such as endoscopic third ventriculostomy (ETV), ventriculoperitoneal shunt (VPS), external ventricular drainage (EVD), or direct resection of the lesion without other diversion methods [7–10]. However, each of these strategies has risks and benefits associated with the therapeutic decision made for each patient, and part of the difficulty in choosing a safer treatment method is due to the multiple outcomes selected in studies with different methodologies, making comparison between studies and between hydrocephalus treatment methods very difficult.

Thus, based on the collaborative effort presented, we identified the need for a prospective multicenter study focusing on the main treatment strategies for hydrocephalus secondary to posterior fossa tumors in children. The PITCH study aims to compare different treatment modalities for obstructive hydrocephalus secondary to posterior fossa tumors (endoscopic third ventriculostomy, external ventricular drainage, ventriculoperitoneal shunt, and tumor resection), considering the number of surgeries related to hydrocephalus treatment and its associated complications within the first year after the initial procedure performed to treat hydrocephalus, as well as mortality and infection rates.

## Methods

A prospective, multicenter, controlled cohort study will be conducted. The primary outcome will be the number of surgical interventions related to the treatment of hydrocephalus during the follow-up period, as it directly reflects treatment failure and holds significant clinical relevance. Secondary outcomes will include mortality, infection, and other clinically relevant complications, which will be analyzed as complementary endpoints. The study was approved by the Scientific Committee of the Instituto de Oncologia Pediátrica (IOP/GRAACC) and by the Ethics Committee of the Universidade Federal de São Paulo (UNIFESP), as well as by the ethics committees of all participating centers. Patients will be included in the study after obtaining written informed consent from their legal guardians and assent from patients under 18 years of age diagnosed with hydrocephalus secondary to posterior fossa tumors, confirmed by imaging studies (computed tomography (CT) and magnetic resonance imaging (MRI)), and who will undergo surgical resection of the posterior fossa lesion.

### Characteristics of the inclusion and exclusion criteria

The patients included will be under 18 years old with a diagnosis of hydrocephalus secondary to a primary tumor of the central nervous system (CNS) located in the posterior fossa, characterized by clinical data and radiological imaging (CT or MRI).

Hydrocephalus secondary to posterior fossa tumor will be characterized when the patient presents a tumor in the posterior fossa causing obstruction of cerebrospinal fluid circulation and enlargement of the lateral ventricles and the third ventricle, with such enlargement documented by imaging exams such as cranial CT or MRI, which must identify, in addition to ventricular enlargement, the presence of enlargement of the temporal horns of the lateral ventricles (> 2 mm) and transependymal edema in the frontal and/or occipital horns of the lateral ventricles. In addition, the patient must present signs and symptoms related to increased intracranial pressure (ICP), such as headache, irritability, vomiting, strabismus and/or unilateral or bilateral sixth cranial nerve palsy, papilledema, bradycardia, arterial hypertension, and altered level of consciousness (drowsiness, stupor, or coma).

Participating centers will be those that offer, through the public health system and/or the supplementary health system, surgical treatment for patients under 18 years of age with posterior fossa tumor. Patients who were initially treated for hydrocephalus in an external center may be included, provided that all necessary information for inclusion in the study is available.

Patients will be excluded from the study if they have lesions in sites other than the posterior fossa as the main focus, patients who have previously undergone surgical resection of posterior fossa tumors, patients without hydrocephalus associated with posterior fossa tumor, patients with posterior fossa tumors and hydrocephalus who will not undergo surgical resection of the lesion (for example, diffuse intrinsic pontine glioma), or cases in which only biopsy of the lesion is considered, patients who refuse to participate in the study, and patients whose data regarding hydrocephalus and its characterization cannot be recovered with the level of detail necessary for the study.

## Results

Participant recruitment will occur through spontaneous demand and may take place in the immediate postoperative period of the hydrocephalus surgery, but it must occur, without exception, in the preoperative period for the tumor surgery, requiring the signing of the Informed Consent Form. Data collection will occur according to the child’s routine clinical care.

### Outcome assessment

The primary outcome of this study will be the number of reintervention surgeries for the treatment of hydrocephalus after the index surgery for hydrocephalus treatment, which will be chosen at the discretion of each participating center and the attending physician responsible for the patient’s initial treatment.

**Fig. 1:**
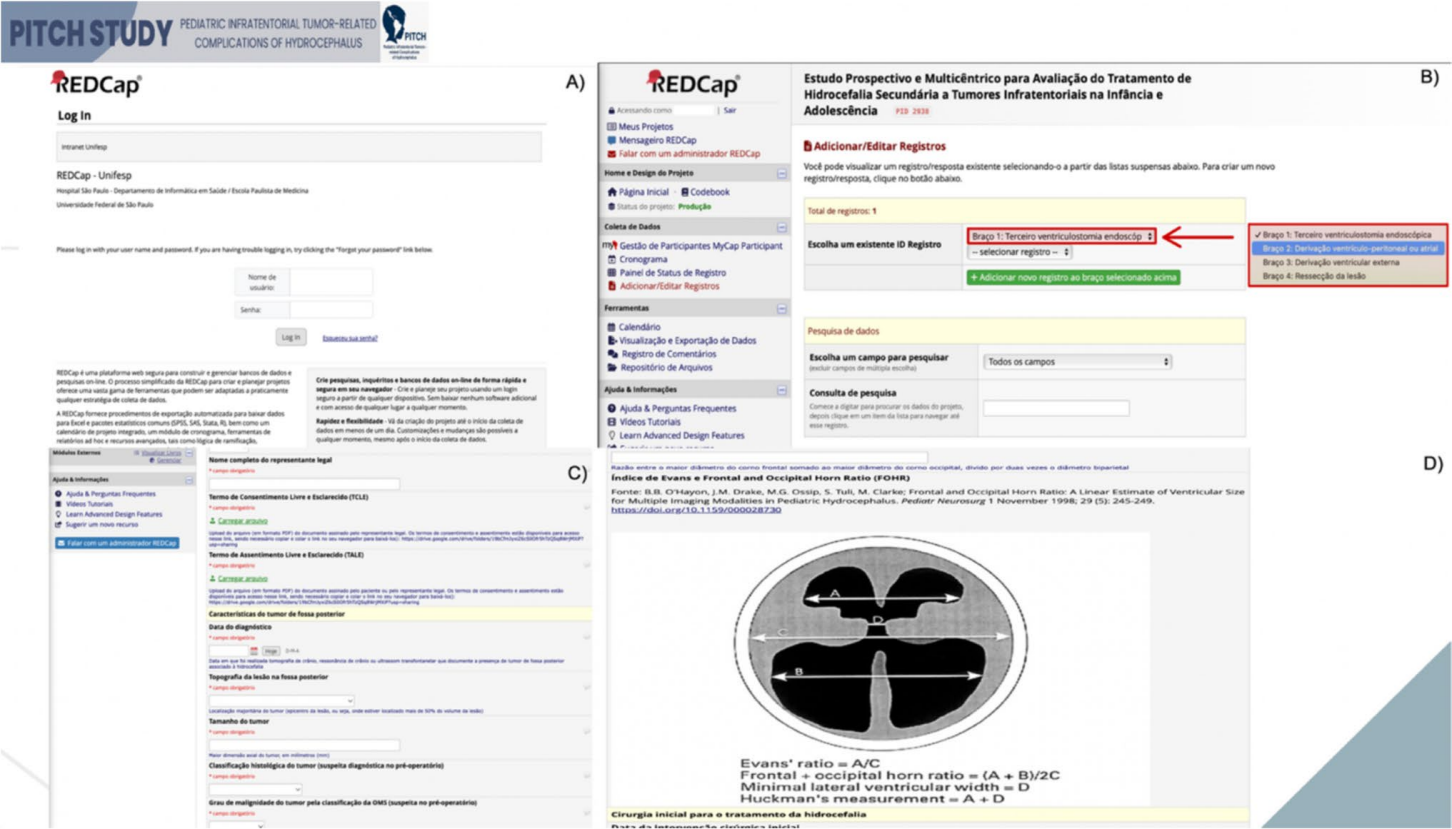
Representative screenshot of the online registry (https://redcap.unifesp.br/), referring to the general data entry and the patient’s inclusion in the study. **A** Login and password that each center receives to begin the data entry. **B** Selection of the research arm. **C**, **D** An overview of the data used

Patients will be included after signing the Informed Consent Form and/or Assent Form and meeting the inclusion and exclusion criteria. Their data will be anonymously entered into the REDCap software (Fig. 1) for prospective follow-up. Each participating center will be recognized by a specific identifying number. Once the patient is selected to participate in the study, they will be prospectively followed, and each outcome event may be recorded in real time or at the predetermined follow-up times of 1 month postoperatively, 3 months, 6 months, 9 months, and 12 months, always considering day zero as the day on which the hydrocephalus treatment was performed.

A separate confidential record will be maintained with the identifying information—full name of the patient and legal guardian, date of birth, sex, ethnicity, identifying number of the participating center, local hospital record, and REDCap ID—to ensure that duplication of patients in the study does not occur.

In the preoperative survey, the following information will be collected (Table 1).

**Table 1:** Information on preoperative variables

Variable	Definition/categories
Patient identification number	Unique patient code
Age	In years
Date of birth	dd/mm/yyyy
Date of diagnosis	Date of the initial exam (CT, MRI, or transfontanellar ultrasound) documenting posterior fossa tumor + hydrocephalus
Lesion topography	Main location (> 50% of volume): • 4th ventricle • Cerebellar hemisphere • Cerebellar vermis • Cerebellopontine angle • Brainstem
Tumor size	Largest axial diameter (mm)
Histological classification	Preoperative diagnosis (if available) + pathological confirmation (e.g., astrocytoma, ependymoma, medulloblastoma, ATRT)
Malignancy grade	WHO 2021 classification (adjusted after final pathology report)
Clinical signs/symptoms	Headache, vomiting, dizziness, gait disturbance, diplopia, facial palsy, choking, etc.
Leptomeningeal spread	Leptomeningeal thickening/enhancement on contrast-enhanced MRI
Hydrocephalus on imaging	Radiological criteria: • Increased Evans index • Temporal horn dilation (> 2 mm) • Ependymal transudation • Ballooning of the 3rd ventricle • “Beaten silver” appearance • Empty sella • Concavity of the 3rd ventricle floor
Type of initial intervention	ETV, EVD, VPS, or tumor resection + date of procedure

For each procedure chosen as a management strategy for hydrocephalus, its particularities will be considered (Table 2).

**Table 2:** Information on the modalities chosen for the treatment of hydrocephalus secondary to posterior fossa tumor

Procedure	Specific variables
VPS	• Location of proximal catheter (frontal/occipital) • Type of valve (fixed, adjustable, flow-regulated) • Brand (national/imported) • Antibiotic impregnation (yes/no) • Distal catheter trimming (yes/no)
EVD	• Use (pre- or intraoperative) • Location of proximal catheter (frontal/occipital) • Brand (national/imported) • Antibiotic impregnation (yes/no) • Initial height of the system
ETV	• Type of endoscope (rigid/flexible) • Satisfactory stoma (opening of the 3rd ventricle and Liliequist membrane, visualization of the basilar artery) • Opening instrument (Fogarty 3F/4F/5F, scissors, biopsy forceps)
Tumor resection	• Surgical approach: midline suboccipital, retrosigmoid, or far-lateral • Without pre/intraoperative implantation of drainage devices

During postoperative follow-up, data will be collected regarding the occurrence of postoperative complications associated with the initial surgical procedure (if any) (Table 3).

**Table 3:** Postoperative information on complications associated with the surgical procedure

Outcome	Definition/criteria	Classification
CSF leak	CSF leakage through the surgical wound (posterior fossa/ETV) • *Simple:* resolves spontaneously or with simple measures (pressure bandage, lumbar puncture, suture reinforcement) • *Clinically important:* continuous leakage, need for dural/skin re-approach or placement of EVD/VPS	Simple/important
Pseudomeningocele	Palpable, clinical, or radiological subdermal CSF collection • *Simple:* improves with puncture/bandaging • *Clinically important:* does not improve, causes significant discomfort, wound risk, or requires surgery/EVD/VPS	Simple/important
Surgical site infection	ANVISA 2017 criteria • *Superficial incisional:* up to 30 days, skin/subcutaneous tissue, criteria of purulent drainage, positive culture or clinical signs • *Deep incisional:* up to 30–90 days (with implants), involves fascia/muscle, criteria of drainage, abscess, dehiscence, fever/local signs	Superficial/deep
Meningitis/ventriculitis	ANVISA 2017 criteria. Positive CSF culture OR clinical signs (fever, headache, neck stiffness, irritability) + laboratory/imaging/microbiology changes	Present/absent
Brain/subdural/epidural abscess/encephalitis	ANVISA 2017 criteria. Positive culture, surgical, histopathological or radiological finding; neurological symptoms and positive microbiology	Present/absent
Hyponatremia	Na + < 135 mg/dL or below laboratory reference, requiring intervention (fluid restriction/NaCl)	Present/absent
Hypernatremia	Na + > 145 mg/dL or above laboratory reference, requiring intervention (DDAVP)	Present/absent
Subdural hygroma	CSF collection over the cerebral convexity, below the dura, on imaging	Present/absent
Intracranial hemorrhage	Positive imaging finding • *Simple:* no clinical repercussions, no surgery • *Clinically relevant:* neurological symptoms + need for surgery	Simple/relevant
CSF hypotension	Orthostatic headache, improvement when lying down + imaging findings (small ventricles, collections, venous engorgement)	Present/absent
Histological classification	WHO 2021 – Tumor malignancy grade	According to WHO
Extent of resection	• Total: no residual tumor • Near-total: < 1.5 cm^3^ • Subtotal: > 1.5 cm^3^	Total/near-total/subtotal
Adjuvant treatment	Record of start date (CT and/or RT)	Date
Reinterventions/additional exams	Need for additional exams or subsequent surgeries, with documentation of type, date, reason, and hospitalization time	Details
Death	Date and relation to hydrocephalus or tumor	Date
Tumor surgery date	When different from initial hydrocephalus treatment	Date
Time intervals	Between initial treatment, tumor surgery, and start of adjuvant therapy	Days/months
Failure of initial treatment	Need for new surgeries for hydrocephalus or related death	Time to failure
Ascending transtentorial herniation	Imaging evidence or neurological worsening after EVD	Present/absent
New tumor hemorrhage	New hemorrhagic focus after EVD/VPS/ETV (imaging or clinical)	Present/absent
Qualitative assessment of the operative field	0–10 scale for two aspects: • Neural relaxation (0 = no relaxation, transdural herniation; 10 = complete relaxation, adequate space for manipulation) • Cerebellar quality (0 = extremely friable/bleeding; 10 = not friable, not bleeding)	Score (0–10)
Cerebellar mutism/posterior fossa syndrome	Diagnosis when criteria A and B1 (mutism) are present, or, in the absence of B1, when A + B2 + C or D are fulfilled Criterion A: Acquired cerebellar lesion (e.g., postoperative or stroke) with symptoms in the following criteria, appearing up to 2 weeks after injury Criterion B: Speech/language deficits: •*B1 Mutism:* total inability to speak •*B2 Impaired language:* short phrases (single words or 2–3-word phrases), agrammatism, atypical rhythm (slow, scanning, ballistic) and/or anomia. Criterion C: Emotional/affective changes: irritability, emotional lability, or flat affect. Criterion D: Motor dysfunction: apraxia, ataxia, dysmetria, hypokinesia, or hemiparesis	Present/absent

## Sample size calculation

The sample size calculation was performed considering the number of surgeries as the primary outcome, which is the main variable of interest in this clinical study. The primary analytical objective is to compare the effect of four treatment strategies on this outcome, with adjustment for age as a relevant covariate.

For sample size estimation, G*Power software version 3.1.9.6 [11] was used, as described by Kang [12], adopting the *F*-test family, specifically the ANCOVA option: fixed effects, main effects, and interactions, in an a priori analysis. This approach is widely used for comparisons between groups with adjustment for covariates in clinical studies [13–15]. A medium effect size (*f* = 0.25), a significance level of 5% (*α* = 0.05), and a statistical power of 80% (1 − *β* = 0.80) were assumed, as recommended to ensure adequate ability to detect clinically relevant differences. The model included four comparison groups and one covariate (age). Based on these parameters, a minimum total sample size of 179 participants was estimated. Assuming a 10% loss to follow-up, the total sample size would be approximately 200 participants, corresponding to about 50 individuals per group, resulting in a test with degrees of freedom *F*(3,174) and a statistical power of approximately 80%.

## Statistical analysis

The statistical analysis of the primary outcomes and the quantitative variables will be compared using the *F*-test family, specifically the ANCOVA. For secondary outcomes, multivariable regression models will be used to adjust for relevant covariates, including demographic, clinical, and radiological variables associated with both treatment selection and outcomes. We will opt for parametric tests when the samples prove to originate from a population with a normal distribution according to the Kolmogorov–Smirnov test. We will consider *α* = 0.05 to characterize statistical significance. Data analysis and graph creation will be performed using GraphPad Prism version 10.1.1 for Mac, GraphPad Software, Boston, MA, USA, and using IBM SPSS Statistics, version 29.0 - 2022 (IBM Corp., Armonk, NY, USA).

## Discussion

The discussion regarding the safest and most efficient drainage method, and when to perform it (before, during, after tumor resection, or whether to simply monitor hydrocephalus after resection without the need for perioperative diversion), remains controversial in the literature [16–19], since each method presents characteristic advantages and disadvantages that also depend on hospital infrastructure [7].

Several studies reported in the literature are restricted to a single center [3, 7, 8, 17–21], and most are limited by the small number of participants [22]. Thus, multicenter, prospective, and randomized studies in this area are necessary [9, 16, 22].

Described in 1923 by William Mixter [23], endoscopic third ventriculostomy provides the benefits of treating hydrocephalus at presentation and reducing its incidence after resection, in addition to avoiding the placement of drains and enabling tumor resection under low intracranial pressure conditions [3, 6].

Moreover, the procedure reduces intraoperative and late complications associated with EVD and VPS placement and promotes the “physiological” restoration of cerebrospinal fluid circulation [21]. However, it is a procedure that may be associated with complications such as hemorrhage, bradycardia, hemiparesis, memory dysfunction, diabetes insipidus, epilepsy, infection, and fistula [21].

Tamburrini G et al. [24], in a study conducted with 30 children, reported that factors such as extent of tumor resection, degree of hydrocephalus, histology, and tumor location were not associated with ETV success. Srinivasan et al. [8] recommended the procedure only in cases of symptomatic hydrocephalus and reported a higher failure rate associated with ependymoma.

VPS is one of the alternatives for the treatment of hydrocephalus [25]. However, it may present complications such as infection, ascites, visceral perforation, hemorrhage, and peritonitis [21], as well as other rarer complications such as device migration or extrusion [26].

Metastatic cell dissemination, such as in medulloblastoma, may occur through the VPS catheter [27–29]. In patients under 19 years of age with hydrocephalus secondary to posterior fossa tumor, ETV failure occurs earlier when compared to VPS, although cumulative failure is lower with ETV (21%) than with VPS (29%) [30].

In the study conducted by El-Gaidi et al. [17], after VPS placement, 121 (84.6%) of the 214 patients improved from symptoms related to increased ICP, 27 (12.6%) showed no changes after VPS, and 6 deteriorated neurologically. Among the 87 patients who underwent ETV, only 60 (67%) presented ICP improvement.

Regarding EVD, we know that it instantly reduces ICP and enables its monitoring visually, without the uncertainty resulting from other temporizing procedures [7], and is used for rapid control of elevated ICP [23]. However, EVD use is considered a risk factor for permanent hydrocephalus [9] and is correlated with complications.

Helmbold LJ et al. [29] correlated the procedure with the occurrence of wound dehiscence and CSF leakage, while Verhey LH et al. [11] reported a higher incidence of postoperative cranial nerve deficits and contraindicated the routine use of intraoperative adjunctive EVD in patients with stable hydrocephalus. Furthermore, in the study by Krause et al. [9], EVD use carried a threefold risk for subsequent ETV or VPS in children older than 2 years and was not shown to be more effective than surgery alone for controlling ventricular enlargement.

Hedrich C et al. [7], when analyzing 114 patients retrospectively, found low rates of infection (lower than those reported in the literature), CSF leakage, and absence of wound dehiscence and pseudomeningocele, thus considering EVD a safe and effective procedure. Helmbold LJ et al. [29] did not find a significant correlation between the procedure and meningitis or pseudomeningocele. Patient selection for EVD placement remains challenging and must be clarified through prospective multicenter analyses [16].

Although EVD is used as a temporary form of treatment, we decided to allocate it as an equivalent arm of the study because it is frequently used as an initial treatment strategy and may directly influence clinical evolution, the occurrence of complications, and the need for subsequent interventions. We believe that its inclusion as a comparator group is justified, as the initial management decision may significantly impact outcomes.

The main limitation of this study will be the lack of randomized treatment allocation. Given the ethical and practical constraints of the clinical setting, randomization was not feasible, which may introduce selection bias. To mitigate this bias, a pre-specified statistical analysis plan was established, including the use of multivariable regression models to adjust for relevant covariates, such as demographic, clinical, and radiological variables associated with both treatment selection and outcomes. In addition, propensity score–based methods will be employed, which may be applied through matching, weighting, or covariate adjustment, depending on statistical appropriateness. Despite these strategies, residual confounding cannot be completely excluded, which remains an inherent limitation of observational studies. Another limitation relates to the use of the operative field quality scale (0–10), which is not a previously validated instrument. This variable was included as an exploratory measure to capture the surgeon’s intraoperative perception of surgical field conditions, which are difficult to quantify using standardized tools. Therefore, its results should be interpreted with caution, and no formal validation or reproducibility testing of this scale was performed.

The present study aims to compare the clinical outcomes associated with the four management strategies analyzed (ETV, VPS, EVD, and primary tumor resection) in a real-world clinical setting. No superiority of any strategy is assumed prior to data analysis, and the study is conducted under the principle of clinical equipoise, with the objective of identifying potential differences in outcomes and contributing to the determination of the most appropriate therapeutic approach.

## Data Availability

No datasets were generated or analysed during the current study.
